# Knowledge about child birth and postpartum obstetric danger signs and associated factors among mothers in Dale district, Southern Ethiopia

**DOI:** 10.1186/s12884-020-02989-7

**Published:** 2020-06-01

**Authors:** Abayneh Desalegn Dangura

**Affiliations:** School of Public health, Adama Science and Technology University, Armauer Hansen Research Institute (AHRI), Addis Ababa, Ethiopia

**Keywords:** Postpartum, Childbirth, Danger signs

## Abstract

**Background:**

Globally, every day, approximately 800 women die from preventable causes related to pregnancy and childbirth. The majority of these deaths occur after childbirth (post-partum period) mostly within 24 h. Raising awareness of women on obstetric danger sign of childbirth and postpartum, are crucial for safe motherhood initiative and to reduce maternal mortality.

**Methods:**

A community based cross sectional study was conducted from December 15, 2017 up to February 10, 2018 on randomly selected sample of 782 women who had at least one delivery in the last 12 months. Multi stage sampling technique was used to select the study participants. Pre tested structured questionnaire was used to collect quantitative data. Bivariate and multivariate logistic regression analyses were performed using SPSS version 20.0 software.

**Results:**

Total 732 women who had at least one birth prior to this survey were interviewed and making a response rate of 93.6%.The most common spontaneously mentioned danger signs during childbirth was Severe vaginal bleeding by 281 (68.4%). Women who could mention at least two danger signs during child birth and post-partum period were 333 (45.5%), 213(29.1%) respectively. Being urban (AOR = 3.54, 95% of CI: [2.20–5.69] and delivered previous birth at health institution (AOR = 3.35, 95% of CI: [2.38–4.72]) were factors found to be significantly associated with knowledge of danger signs during postpartum. Being Attended secondary level and above (AOR = 2.41, 95% of CI: [1.02–7.76]) and use of ANC during last pregnancy (AOR = 3.63, 95% of CI: [2.51–5.25]), were factors found to be significantly associated with knowledge of danger signs during childbirth.

**Conclusions:**

The level of knowledge about danger signs of child birth and postpartum were low. This indicates that many mothers are more likely to delay in deciding to seek health care. Also, knowledge about danger signs of childbirth and postpartum were affected by place of residence, formal education, use of ANC and place of delivery. Therefore, the identified gap in awareness should be addressed through effective maternal health services by strengthening and designing appropriate strategies including provision of targeted health information, education and communication.

## Background

Globally, every day, approximately 800 women die from preventable causes related to pregnancy and childbirth. The majority of these deaths occur after childbirth (post-partum period), mostly within 24 h. Maternal deaths during pregnancy and at the time of delivery contribute about 25 and 15%, respectively [1, 2]. Women in less developed countries have, on average, many more pregnancies than women in developed countries and their lifetime risk of death due to pregnancy is higher [1, 2]. Maternal death is undesirably high and about 295,000 women died during pregnancy and childbirth in 2017. The vast majority of these deaths in high-income countries is 1 in 5400 and 1 in 45 in low income countries, and most could have been prevented [3–5].

Most maternal deaths are avoidable, as the health-care solutions to prevent or manage complications are well known. All women need access to high standard of care in pregnancy, and during and after childbirth. Women’s health and newborn health are closely integrated. It is particularly important that all births are attended by skilled health professionals, as timely management and treatment can make the difference between life and death for the mother as well as for the baby. Beside of this, because of these women stricken during their most productive years, their deaths have a profound impact on the society and on the economies of their nations at large. Most of maternal deaths in under developing countries including Ethiopia was due to adequate heath care system and family planning, and pregnant women have minimal access to skilled labor and emergency care [6]. As many studies indicate that about 50% of maternal deaths are due to post-partum hemorrhage [2, 3, 6, 7]. The danger signs are not the actual complications, but symptoms that are easily identified by non-clinical personnel. The commonest/ key danger signs during labor and child birth include severe vaginal bleeding, prolonged labor (greater than 12 h), convulsion, and retained placenta. Major danger signs during the postpartum period include severe vaginal bleeding, foul smelling vaginal discharge, and fever [7, 8].

Preventing maternal mortality has arrived at priority of health and development agendas. One way of reducing maternal mortality is by improving availability, accessibility, quality and use of services for treatments of complications that arises during pregnancy, childbirth and post-partum period [7, 9–11]. Most of maternal deaths are avoidable, if women and their families aware about obstetric danger signs and all women need access to high quality care in pregnancy, during and after childbirth. The evidence suggests that raising awareness of women about danger signs of childbirth and postpartum period would improve early detection of problems and reduces delay in the deciding to seek obstetric care [9, 12, 13].

Similarly, because of most babies are born at home or are discharged from the hospital in the first 24 h, increasing community awareness of the danger signs of pregnancy and newborn complications is of critical importance for improving newborn survival [7, 9, 14, 15]. Thus, this has been identified as one of the key strategies for improving maternal and child health [9, 16, 17]. However, like in many developing countries, awareness of women about danger signs of childbirth and postpartum remains low in Ethiopia [18–22]. This study therefore aims to fill this gap by assessing the current status of knowledge about danger signs of childbirth and postpartum among mothers in the study area.

## Methods

The study was conducted in Dale district of Southern Ethiopia, which was one of 19 woreda of Sidama zone, SNNPR region, Ethiopia and which is located 308 km Southeast of Addis Ababa. Dale is one of 19 Districts in Sidama Zone of South Nations and Nationalities Regional State. As projected from the 1994 Ethiopian census, the district had a total population of 264, 544 and 61,639 women of age 15–49 years [23]. Administratively, the District was subdivided into 36 rural and 6 urban Kebeles in which 264,544 and 45,528 population respectively. The study was conducted from December 15, 2017 up to February 30, 2018. Community based cross-sectional study was conducted to measure the level of knowledge about danger signs of childbirth, postpartum and associated factor among mothers who gave birth in the last 12 months prior to the survey on randomly selected kebeles in Dale district of southern Ethiopia.

### Sample size and sampling procedure

The required sample size was determined using Statcalc program of the EpiInfo statistical package, which was estimating a single population proportion with the assumption that the proportion of knowledge about the obstetric danger signs (36.4%) [18], 5% of margin of error, 95% of confidence interval and design effect of 2, which gives 711 study participants and 10% of expected non response rate, which gives sample size of total 782 study participants. Multistage sampling was used to select the study subjects. First, all the kebeles/ sub-districts in the district were stratified into urban and rural. Then 2 out of 6 urban and 11 out of 36 rural Kebeles were randomly selected. The calculated sample size was proportionally allocated to urban (*n* = 120) and rural (*n* = 662) according to their number of households. Then, sampling frames of households was prepared for each kebele in collaboration with the administrators of respective kebeles. Households with a woman who gave birth in the last 12 months prior to the survey were selected and grouped into the village and the villages were selected by using simple random sampling. For selecting the study participants, eligible women who found in the selected villages were the part of the study until sample size allocated for each kebele was enough. Whenever more than one eligible respondent were found in the same selected household, only one respondent was chosen by lottery method.

### Data collection procedure and data quality control

Pretested and structured interviewer administered questionnaire, which was first prepared in English and translated into local language (Sidamigna) was employed to obtain information on socio-demographic, obstetric history, and knowledge of women about danger signs of childbirth and postpartum. Diploma clinical nurse interviewers, they were fluent in the local language (Sidamigna) and familiar with the local customs, were employed to collect the data and supervised by one bachelor degree graduated nurse professional and one bachelor degree graduated Public health professional who were currently working in the catchment health centers and by the principal investigator as well. The data collectors and supervisors were trained for 1 day on data collection.

The questionnaire was pre-tested on 39 mothers who gave birth in the last 12 months in Wonsho district to assess for its clarity, length, and completeness and skip patterns. Then after some adjustment was done in the questionnaire and extra briefing were given to the data collectors and supervisors. To insure the quality of the data, daily meeting was held between the principal investigator and data collectors to troubleshoot any problems that arose. In addition, inspection for completeness and quality of data collection was carried out daily by the supervisors and detailed feedback was provided to data collectors.

### Data quality assurance

Questionnaire was prepared in English version and translated in to Sidamigna local language and back to English. It was pre-tested on 5% of the calculated sample size in Wonsho district that was not select in the study. Additional adjustment on questionnaire was made based on the results of the pre-test. Data collection was carried out by trained nurses who were selected from the catchment health facilities. Ten percent of the collected data was check by the supervisor daily for completeness and finally the principal investigator monitored the overall quality of data collection.

### Data processing and analysis

The collected data was cleaned, coded, and entered into Epi Info and then exported to SPSS version 20.0 window program for further analysis. Frequencies and cross tabulations were used to check for missed values and variables. Errors were identified and corrected after revising the original questionnaires. Frequencies, proportions, measure of central tendency and measure of variation were used to describe the study subjects. Descriptive statistics was used to measure level of knowledge about danger signs of childbirth, postpartum and frequencies of respondents’ socio demographic characteristics. Multivariate and bivariate logistic regressions were computed to identify associated factors of knowledge about danger signs of childbirth and postpartum. Odds ratio was calculated both to assess the association and measure the strength of the association between explanatory and outcome variables. Finally, the results were presented using crude odds ratio (COR), adjusted odds ratio (AOR) and confidence level (95% CI). In all analyses, *P* value < 0.05 were considered as a level of significance.

### Operational definition

**Spontaneous knowledge:** refers to the respondent’s naming a sign without being asked about that sign by name.

**Knowledgeable about danger signs of childbirth** Women who can spontaneously mentioned at least two danger signs of childbirth from seven danger signs.

**Knowledge about danger signs of postpartum:** Women who can spontaneously mentioned at least two danger signs of postpartum from ten danger signs.

**Danger signs**: refers to the alert of obstetric complications those occur commonly in the middle and late pregnancy, labor/child birth and post-partum period. The danger signs that was looked at in this study include severe vaginal bleeding, convulsions, severe headache, blurred vision, severe abdominal pain, high fever, loss of consciousness, labor lasting greater than 12 h, accelerated/reduced fetal movement, swelling of fingers, face and legs [2, 7, 24].

### Ethics approval and consent to participate

Ethical clearance was obtained from research ethical clearance board of Addis continental institute of public health and Adama science and Technology University, and permission and support letter was obtained from Dale woreda health office. Before enrolling any of the eligible study participants, the purpose, the benefits, and the confidential nature of the study was described and discussed for each participant. Written informed consent was obtained from all participants. In the case of age less than 18 years verbal consent was obtained from their family and approved by ethics review committee. The discussions between the data collectors and the respondents were takes place privately and individually. Only those consented and proved their willingness to take part in the study was enrolled and interviewed.

To keep confidentiality, the information collected from this research project was kept confidential and information from the participant was collected by this study was stored in the file, without participant’s name. In addition, it was not revealed to anyone except the investigator and all responses given by participants was kept confidential by using key and locked system like computer pass word whereby no one have an access to see it and at the end of the data analysis and thesis presentation, the questionnaire was burned. The women who had no knowledge about danger signs of pregnancy, health information was given about possible danger signs that can occur during childbirth and postpartum and if any one of danger sign happens, they should have go to health institution urgently and report immediately to health professionals to obtain emergency obstetric care.

## Result

### Socio demographic profile of the respondents

Total 732 women who had at least one birth prior to this survey were interviewed making the response rate of 93.6%. Two hundred seventy four (37.4%) were between the age group of 20–24 years with the mean age of 24.8 + 2.9 standard deviation. The place of residence of the majority respondents accounts of 621 (84.8%) were rural dwellers, 707 (96.6%) currently in marital union, 595 (81.3%) protestant of their religion, 653 (89.2%) Sidama by ethnicity, 292 (39.9%) unable to read and write, 620 (84.7%) were house wives, and 425 (58.1%) of monthly income less than 10 US dollar. Regarding to husband’s profile of the total respondents from 707, 239 (33.8%) were between the age group of 30–34 years with the mean age of 30.7 + 3 standard deviation, 195 (27.6%) unable to read and write, 197 (27.9%) attained secondary level of education, 474 (67%) were farmer and 357 (50.5%) had monthly income of less than 30 US dollar. The mean number of family members in the household was 4.7 + 1.2 standard deviation, 452 (61.7%) had functional radio/television in their home and 502(68.6%) were less than 1 h of time taken to nearby health facilities (Table 1).

**Table 1:** Socio demographic characteristics of mothers in age of 15–49 years in Dale district, Southern Ethiopia, March 2018, (*n* = 732)

Category of variables	Sub category	Frequency	Percent (%)
Place of residence (*n* = 732)	Rural	621	84.8
	Urban	111	15.2
Age of the mothers (*n* = 732)	15–19years	28	3.8
	20–24years	274	37.4
	25–29years	225	30.7
	30–34 years	168	23.0
	> 35 years	37	5.1
Current marital status of the mother (*n* = 732)	marital union	707	96.6
	not marital union	25	3.4
Religion of mother (*n* = 732)	Protestant	595	81.3
	Orthodox	57	7.8
	Muslim	58	7.9
	Catholic	22	3.0
Ethnic group of mother (*n* = 732)	Sidama	653	89.2
	Amhara	49	6.7
	Oromo	8	1.1
	Guraghe	16	2.2
	Others(specify)^a^	6	0.8
Educational level of mother (*n* = 732)	Unable to read and write	292	39.9
	Read and Write	168	23.0
	Primary education	185	25.3
	Secondary education	69	9.4
	College and above	18	2.5
Occupation of the mother (*n* = 732)	Housewife	620	84.7
	merchant	7	1.0
	Government employee’s	23	3.1
	Private gainful worker	82	11.2
Monthly income in USD (*n* = 732)	< 10	425	58.1
	10–20	189	25.8
	20–40	66	9.0
	> 40	52	7.1
Age of the husband (*n* = 707)	15-24 Years	56	7.9
	25–34 years	468	66.2
	> 35 years	183	25.9
Educational level of the husband (*n* = 707)	unable to read and write	195	27.6
	Primary education	262	37.0
	Secondary education	197	27.9
	College and above	53	7.5
Occupation of the husband (*n* = 707)	Farmer	474	67.1
	Government employee	160	22.6
	Others^b^	73	10.3
Monthly income in USD (*n* = 707)	< 20	357	50.5
	20–40	196	27.7
	> 40	154	21.8
Family Size (*n* = 732)	1–3	311	42.5
	4–6	328	44.8
	> 6	93	12.7
Had functional radio/television in the house hold (*n* = 732)	Yes	452	61.7
	No	280	38.3
Time taken to nearby health facilities (*n* = 732)	< 1 h	502	68.6
	> = 1 h	230	31.4

Note: Others ^a^Siltie, Hadiya, Kanbata, Gamo Gofa

^b^Daily workers, Students, merchants

### Maternal and obstetric characteristics of the respondents

Out of 732 the respondents, 438 (59.8%) were between the 20–29 years of age group at first pregnancy with the mean age at first pregnancy 19.5 + 2 standard deviation, 308 (42.1%) had 3 and more number of pregnancies with the mean number of pregnancy of 2.2 + 0.9 standard deviation, and 87 (11.9%) of respondent had history of still birth. Regarding to maternal health services utilization, 551 (75.3%) were used antenatal care during the last pregnancy. Among those who attended antenatal care, 169 (30.7%) had made four visit and more, and 85 (15.4%) of respondents had made only one visit of antenatal care during the last pregnancy. From those who attended antenatal care (551), 107 (27.4%) of women were informed about danger signs and can mention at least one danger sign with the source of information obtained during antenatal care. Among those who delivered at health institution 76 (27.2%) were informed about danger signs and can mention at least one danger sign with source of information from health education given during delivery at health facilities. The final decisions made on the place of delivery by their own selves were 579 (79.1%) (Table 2).

**Table 2:** Maternal and obstetric characteristics of the respondents in Dale distinct, Southern Ethiopia, March 2018 (*n* = 732)

Category of variables	Sub category	Frequency	Percent (%)
Age at first pregnancy	15–19 years	293	40.0
	20–29 years	438	59.8
	30 and above	1	0.14
Gravida (total number of pregnancy)	1	208	28.4
	2	216	29.5
	3 and above	308	42.1
Para (total number of gave birth)	1	208	28.4
	2	216	29.5
	3 and above	308	42.1
total number of Live birth	1	152	23.6
	2	202	31.4
	3 and above	289	45.0
Total number of still birth	0	643	87.8
	1	87	11.9
	2	2	0.27
	3 and above	0	0
Use of antenatal care during last Pregnancy	Yes	551	75.3
	No	181	24.7
Number of visit antenatal care during last pregnancy	1 visit	85	15.4
	2–3 visit	297	53.9
	4 visit and above	169	30.7
Place of deliver of previous birth	Home	453	61.9
	Health institution	279	38.1
final decision maker on place of delivery during last pregnancy	Self	579	79.1
	Family/Husband	114	15.6
	Relatives	39	5.3

### Knowledge about obstetric danger signs of childbirth and postpartum

Out of 732 respondents, women who had at least one birth prior to this survey could mention at least one danger signs of childbirth by 411 (56.1%), and post-partum period by 269 (36.7%). Among those who mentioned at least one danger signs, the most common spontaneously mentioned danger signs during childbirth were Severe vaginal bleeding by 281 (68.4%), severe headache by 121 (29.4%), convulsion by 113 (27.5%), high fever by 101 (24.6%), loss of consciousness by 81 (19.7%), Labor lasting greater than 12 h by 70 (17.0%) and Placenta not delivered 30 min after delivery by 77 (18.7%).

During postpartum period, the most common spontaneously mentioned danger signs were Severe vaginal bleeding by 117 (16%), Severe headache by 63 (8.6%), convulsion by 51(7%), swollen hand or face by 39 (5.3%), high fever by 50 (6.8%), loss of consciousness by 45 (6.2%), difficult of breathing by 48 (6.6%), severe weakness by 51 (7%) and malodorous vaginal discharge 65 (8.9%). Regarding to knowledge about danger signs of child birth and postpartum; among women who mentioned danger signs of childbirth were 78 (19%) and postpartum 56 (20.8%), could mention only one danger sign (Tables 3 and 4).

**Table 3:** Knowledge of danger signs of labor and childbirth mentioned among women who had at least one birth prior to this survey in Dale distinct, Southern Ethiopia, March 2018, (*n*=411)

Danger signs of labor and child birth	Responses	Frequency	
		Number	Percent (%)
Severe vaginal bleeding	Yes	281	68.4
	No	130	31.6
Severe headache	Yes	121	29.4
	No	290	70.6
Convulsion	Yes	113	27.5
	No	298	72.5
High fever	Yes	101	24.6
	No	310	75.4
Loss of consciousness	Yes	81	19.7
	No	330	80.3
Labor lasting greater than 12 h	Yes	70	17.0
	No	341	83.0
Placenta not delivered 30 min after delivery	Yes	77	18.7
	No	334	81.3

**Table 4:** Knowledge about danger signs of postpartum mentioned among women who had at least one birth prior to this survey in Dale distinct, Southern Ethiopia, March 2018, (*n* = 732)

Danger signs of pregnancy	Responses	Frequency	
		Number	Percent (%)
Severe vaginal bleeding	Yes	117	16.0
	No	615	84.0
Severe headache	Yes	63	8.6
	No	669	91.4
Blurred vision	Yes	51	7.0
	No	681	93.0
Convulsion	Yes	67	9.2
	No	665	90.8
Swollen hands/face*.*	Yes	39	5.3
	No	693	94.7
High fever	Yes	50	6.8
	No	682	93.2
Loss of consciousness	Yes	45	6.2
	No	687	93.8
Diffecult of breathing	Yes	48	6.6
	No	684	93.4
Severe weakness	Yes	51	7.0
	No	681	93.0
Malodorous vaginal discharge	Yes	65	8.9
	No	667	91.1
Others^a^	Yes	2	0.3
	No	730	99.7

^a^Others include excess vomiting, severe abdominal pain

Among the respondents who mentioned two danger signs of labor or child birth by 189 (46%), and postpartum by 109 (40.5%). Also, mentioned three danger signs of child birth by 82 (19%), and postpartum by 68 (25.3%). Those mentioned four danger signs of child birth by 40 (9.7%) and postpartum by 22 (8.2%), and could mention five or more danger signs of child birth by 22 (5.4%) and postpartum by 14 (5.2%). The mean score of mentioned danger signs of childbirth and postpartum were 2.19 and the distribution was approximately normally distributed.

Therefore, those who could mention at least two danger signs of the childbirth were considered as knowledgeable about danger signs of childbirth and those who could mention at least two danger signs of the postpartum were considered as knowledgeable about danger signs of postpartum. Out of 732 respondents, 333 (45.5%) and 213 (29.1%) of women were knowledgeable about danger signs of child birth and postpartum period respectively at 95% of CI (0.39, 0.48).

Mothers who attained their education at secondary level and above were about two times (AOR = 2.41, 95% of CI: [1.02–7.76]) and (AOR = 1.57, 95% of CI: [1.07–9.20]) more likely to have higher knowledge of danger signs of child birth and postpartum period as compared with those who have no formal education. Age of the mothers also increased likelihood of being knowledgeable about danger signs of postpartum was about two times (AOR = 2.06, 95% of CI: [1.02–9.22]) more likely among their age greater than 35 years old. Place of residence had shown strong statistical association with mentioning at least two danger signs. Among women who resident in urban had (AOR = 2.28, 95% of CI: [1.42–3.66]), and (AOR = 3.54, 95% of CI: [2.20–5.69]), more likely to be knowledgeable about danger signs of child birth and post-partum.

### Factor associated with knowledge about childbirth and postpartum danger signs among women

In bivariate and/ or multivariate logistic regression analysis and after adjusted for age, place of residence, education, having functional radio/television in the house, time or distance take to near health facilities, use of antenatal care and place of delivery were independently associated with mentioning of at least two dangers signs of childbirth and postpartum.

Age of mother shows statistical association with mentioned at least two danger signs of Postpartum. Among women whose age was greater than 35 years had about two times (AOR = 2.06 (1.02–9.22) more likely to be knowledgeable as compared with age below 20 years. Level of education shows statistical association with mentioned at least two danger signs of childbirth and postpartum period. Mothers who attained their education at secondary education level and above when compared with rural residents.

Place of delivery and use of antenatal care had also shown strong statistical association with mentioning at least two danger signs of childbirth and postpartum period. Among women who delivered previous birth at health institution had about two times (AOR = 2.42, 95% of CI: [1.69–3.48]) more knowledgeable about danger signs of childbirth and (AOR = 3.35, 95% of CI: [2.38–4.72]) more knowledgeable about danger signs of postpartum as compared to those who delivered at home. Regarding to use of antenatal care, among women who used antenatal care during last pregnancy were (AOR = 3.63, 95% of CI: [2.51–5.25]), and (AOR = 1.72, 95% of CI: [1.26–3.98]) more likely to be knowledgeable about danger signs of childbirth and postpartum respectively as compared with not attained ANC during the last pregnancy.

Having functional radio/TV in the household had shown statistical association. Among women who had functional radio/TV in the household were about two times (AOR = 2.32, 95% of CI: [1.69–3.19]) more knowledgeable about danger signs of childbirth, and (AOR = 2.29, 95% of CI: [1.64–3.21]) more likely to be knowledgeable about danger signs of postpartum as compared with those who haven’t functional radio/TV in the household.

Other predictors of knowledge about obstetric danger signs include distance to nearby health facilities and monthly income of the women. In bivariate analysis, significant association was observed between knowledge about danger signs and distance to health facilities. Among women who where time taken to nearby health facilities less than 1 h had (COR = 1.46, 95% of CI: [1.08–1.99]) more likely to be knowledgeable about danger signs of childbirth as compared to those who far greater than 1 h of health facilities from their living house. Maternal income among the socio economic factors was significantly associated with knowledge of danger signs. Mothers with monthly income greater than 40 US dollar had (COR = 1.90, 95% of CI: [1.07–3.40]) more likely to be knowledgeable about danger signs of postpartum as compared to those who had monthly income less than 10 US dollar. But significant associations of monthly income of the mothers and distance to health facilities were not retained in the multivariate analysis (Table 5).

**Table 5:** Association between knowledge of at least two danger signs of childbirth and postpartum and selected socio demographic and obstetric Characteristics of mothers in Dale district, Southern Ethiopia, March 2018 (*n* = 732)

Variables and sub category	Knowledgeable about danger signs of childbirth				Knowledgeable about danger signs of Post-partum			
	Yes	No	COR (95% of CI)	AOR (95% of CI)	Yes	No	COR (95% of CI)	AOR (95% of CI)
**Age**								
15–19 years	15 (53.6%)	13 (46.4%)	1.00	**1**.00	7 (25.0%)	21 (75.0%)	1.00	1.00
20–24 years	15 (56.6%)	119 (43.4%)	1.13 (0.36–2.44)	0.93 (0.35–2.62)	97 (35.4%)	177 (64.6%)	1.64 (0.78–3.10)	1.73 (0.84–3.55)
25–29 years	12 (57.3%)	96 (42.7%)	1.16 (0.62–2.45)	0.83 (0.41–1.70)	97 (43.1%)	128 (56.9%)	2.27 (0.94–3.47)	1.89 (0.90–3.97)
30–34 years	93 (55.4%)	75 (44.6%)	1.07 (0.54–2.17)	0.80 (0.39–1.63)	51 (30.4%)	117 (69.6%)	1.31 (0.68–2.84)	1.16 (0.57–2.39)
> = 35 years	19 (51.4%)	18 (48.6%)	0.92 (0.34–2.24)	0.78 (0.38–1.62)	17 (45.9%)	20 (54.1%)	2.55 (0.87–7.45)	2.06 (1.02–9.22)*
**Residence**								
Rural	330 (53.1%)	291(46.9%)	1.00	1.00	191 (30.8%)	430 (69.2%)	1.00	1.00
Urban	81 (73.0%)	30 (27.0%)	2.38 (1.52–3.73)*	2.28 (1.42–3.66)*	78 (70.3%)	33 (29.7%)	5.32(3.42–8.27)*	3.54(2.20–5.69)*
**Marital status**								
marital union	402 (56.9%)	305 (43.1%)	2.34 (1.02–5.37)*	2.02 (0.87–4.67)	263 (37.2%)	444 (62.8%)	1.87 (0.74–4.76)	1.44 (0.57–3.68)
Not marital union	9 (36.0%)	16 (64.0%)	1.00	1.00	6 (24.0%)	19 (76.0%)	1.00	1.00
**Education**								
unable to read /write	145 (49.7%)	147 (50.3%)	1.00	1.00	88 (30.1%)	204 (69.9%)	1.00	1.00
read and write	103 (61.3%)	65 (38.7%)	2.25 (0.67–7.56)	1.84 (0.53–6.36)	64 (38.1%)	104 (61.9%)	1.43 (0.93–8.20)	1.37 (0.37–7.87)
primary education	107 (57.8%)	78 (42.2%)	2.55 (0.81–8.05)	1.89 (0.58–6.15)	76 (41.2%)	109 (58.8%)	1.62 (1.03–7.97)*	1.39 (0.23–8.04)
Secondary education	42 (60.9%)	27 (39.1%)	2.21 (0.70–7.00)	1.40 (0.42–4.63)	29 (42.0%)	40 (58.0%)	1.68 (1.16–9.08)*	1.57 (1.07–9.20)*
College & above	14 (77.8%)	4 (22.2%)	3.54 (1.14–11.04)*	2.41 (1.02–7.76)*	12 (66.7%)	6 (33.3%)	4.64 (1.68–12.74)*	2.26 (0.39–13.07)
**Occupation**								
Housewife	340 (54.8%)	280 (45.2%)	1.00	1.00	221 (35.6%)	399 (64.4%)	1.00	1.00
merchant	3 (42.9%)	4 (57.1%)	0.62 (0.15–1.29)	0.38 (0.12–1.19)	2 (28.6%)	5 (71.4%)	0.72 (0.56–1.46)	0.93 (0.57–1.58)
Gov’t employee’s	18 (78.3%)	5 (21.7%)	2.96 (0.84–7.92)	2.16 (0.45–10.17)	15 (65.2%)	8 (34.8%)	3.38 (0.50–12.88)	1.14 (0.31–6.38)
private gainful workers	50 (61.0%)	32 (39.0%)	1.29 (0.80–2.06)	1.24 (0.75–2.06)	31 (37.8%)	51 (62.2%)	1.09 (0.68–1.77)	0.43 (0.10–2.11)
Monthly income in USD								
< 10	224 (52.7%)	201(47.3%)	1.00	1.00	139 (32.7%)	286 (67.3%)	1.00	1.00
10–20	122 (64.6%)	67 (35.4%)	1.71 (0.61–2.51)	1.18 (0.67–2.08)	80 (42.3%)	109 (57.7%)	1.51 (0.73–3.17)	1.41 (0.67–2.98)
20–40	35 (53.0%)	31 (47.0%)	1.01 (0.51–1.44)	0.89 (0.49–1.62)	25 (37.9%)	41 (62.1%)	1.26 (0.68–2.33)	1.12 (0.60–2.11)
> 40	30 (57.7%)	22 (42.3%)	1.22 (0.68–2.19)	1.12 (0.60–2.13)	25 (48.1%)	27 (51.9%)	1.90 (1.07–3.40)*	1.57 (0.87–2.85)
**Parity**								
1	122 (58.4%)	87 (41.6%)	1.00	1.00	77 (36.8%)	132 (63.2%)	1.00	1.00
2–3	131 (61.2%)	83 (38.2%)	1.13 (0.74–1.91)	1.36 (0.95–1.94)	91 (42.5%)	123 (57.5%)	1.20 (0.83–1.74)	1.09 (0.74–1.59)
> 3	158 (51.1%)	151 (48.9%)	0.75 (0.52–1.06)	0.91 (0.62–1.35)	101 (32.7%)	208 (67.3%)	0.79 (0.53–1.17)	0.76 (0.51–1.13)
Having functional radio /TV in the household								
No	121 (43.2%)	159 (56.8%)	1.00	1.00	69 (24.6%)	211 (75.4%)	1.00	1.00
Yes	290 (64.2%)	162 (35.8%)	2.35 (1.73–3.19)*	2.32 (1.69–3.19)*	200 (44.2%)	252 (55.8%)	2.43 (1.75–3.37)*	2.29 (1.64–3.21)*
Time taken to nearby health facilities								
< 1 h	284 (59.4%)	194 (40.6%)	1.46 (1.08–1.99)*	1.23 (0.88–1.70)	175 (36.6%)	303 (63.4%)	0.98 (0.72–1.35)	0.79 (0.57–1.12)
> = 1 h	127 (50.0%)	127 (50.0%)	1.00	1.00	94 (37.0%)	160 (63.0%)	1.00	1.00
Use of ANC during last pregnancy								
No	58 (32.0%)	123 (68.0%)	1.00	1.00	45 (24.9%)	136 (75.1%)	1.00	1.00
Yes	353 (64.1%)	198 (35.9%)	3.78 (2.65–5.41)*	3.63 (2.51–5.25)*	224 (40.7%)	327 (59.3%)	2.07 (1.75–3.37)*	1.72 (1.26–2.98)*
**Number of ANC visit**								
1 visit	43 (50.6%)	42 (49.4%)	1.00	1.00	36 (42.4%)	49 (57.6%)	1.00	1.00
2–3 visit	196 (66.0%)	101(34.0%)	1.89 (0.67–2.83)	1.01 (0.37–2.77)	114 (38.4%)	183 (61.6%)	0.85 (0.56–1.66)	0.99 (0.57–1.72)
4 visit & above	114 (67.5%)	55 (32.5%)	2.02 (0.76–5.68)	1.63 (0.54–4.89)	74 (43.8%)	95 (56.2%)	1.06 (0.72–1.93)	1.11 (0.67–1.83)
**Place of delivery of Previous birth**								
Home	201 (44.4%)	252 (55.6%)	1.00	1.00	116 (25.6%)	337 (74.4%)	1.00	1.00
Health institution	210 (75.3%)	69 (24.7%)	3.81 (2.74–5.30)*	2.42 (1.69–3.48)*	153 (54.8%)	126 (45.2%)	3.53 (2.57–4.84)*	3.35 (2.38–4.72)*
**Final decision maker on place of delivery**								
Self	305 (52.7%)	274 (47.3%)	0.49 (0.28–1.35)	0.62 (0.29–1.34)	195 (33.7%)	384 (66.3%)	0.60 (0.28–1.30)	0.59 (0.27–1.29)
Husband /relatives	106 (69.3%)	47 (30.7%)	1.00	1.00	74 (48.4%)	79 (51.6%)	1.00	1.00

*P-value significant at p < 0.05

## Discussion

The finding of this study revealed that knowledge of danger signs of childbirth and postpartum among mothers. The most commonly mentioned danger sign was severe vaginal bleeding reported by 281 (68.4%) and 117 (16%) during childbirth and post-partum period respectively, which was greater when comparing with study done in Aleta wondo district of Southern region in 2011, (which was 55% during childbirth) [18]. This might be currently government has doing great efforts (like ‘maternal forum’) held at kebele level to increase awareness on danger signs of childbirth and postpartum period, through mobilizing the pregnant mothers to deliver at health institutions by skilled health personnel.

Regarding to knowledge about danger signs, 333 (45.5%) and 213 (29.1%) of women could mention at least two danger signs of childbirth and postpartum. This result was slightly similar with study done in Uganda [25] and study done in Debre Birhan town [26]. However, lower than study done in Tsegedie district of Tigray region [27]. This difference could be variation in socio demographic or cultural characteristics, geographical, health service coverage and use of different sampling techniques.

Education has an effect on knowledge about danger signs of childbirth and postpartum. The educated mothers who completed at least primary education were more knowledgeable about danger signs than uneducated mothers. Similarly, the result was the same with the study carried out in Aleta wondo district [18], Tsegedie district [27], Tanzania [28], Uganda [29], and Egypt [30]. The possible explanations might be related to the fact that educated women have better power to make their own decision in matters related to their health, better access to health service information, improved perception of danger signs or about its complications and can utilize such information optimally. In addition to this, educated women have greater opportunity or access to use health care services.

Age has also an effect on knowledge of danger signs. As respondents’ age increased, the likelihood of being knowledgeable about danger signs also increased. This finding was similar with the study done in Aleta wondo district [18], Tsegedie district [27], Tanzania [28] and Egypt [30]; this is the fact that increased awareness among older women might be related to their own experience of pregnancy and childbirth or complication experienced during previous birth. But different from the study conducted in Arba Minch [31]. This difference might be since the study was conducted in the town, younger women could be more educated, had greater exposure to heard mass media and access to utilize maternal health services than elder women. Place of residence was seen strong statistical association with mentioning at least two danger signs of childbirth and postpartum. Those who resident in urban had about two times more knowledgeable about danger signs of childbirth and about three times more knowledgeable about postpartum when compared with mothers who resident in rural areas. This finding was similar with study conducted in Aleta wondo district [18], Tsegedie district [27], Egypt [30] and Burkina Faso [32]. This could be the fact that urban residents had better access to utilize maternal health service, shorter distance to travel health facilities, had greater exposure to heard mass media and better access to health services information as compared with rural counter parts. Regarding to current marital status, those who were currently in married union had two fold more knowledgeable about danger signs of childbirth when compared with currently in non-marital union. But, this significant association was not retained in multivariate analysis. This finding was similar with the study conducted in Debre Birhan town [26], Tanzania [33] and Egypt [25], but different from Tsegedie district [24]. This needs to be further study and investigation.

Place of delivery and use of antenatal care also strong predictors that affect the knowledge level of women about danger signs of childbirth and postpartum. Mothers who delivered previous birth at health institution had two times more knowledgeable about danger signs of childbirth and postpartum. In addition, mothers who attended at least one visit of antenatal care during last pregnancy were about three times more likely to be knowledgeable about danger signs of childbirth as compared with those who did not attained ANC during the last pregnancy. This finding is in line with the study carried out in Tsegedie district [27], Arba Minch [31], Tanzania [28], Egypt [30] and Nepal [34]. This could be due to heath education and counseling given by heath personnel during utilization of ANC and delivery service. The source of information was asked among women who mentioned at least one danger sign of childbirth and postpartum, 40% of mothers were responds as source of information about danger signs were obtained from heath information given during institutional delivery and use of antenatal care.

Having functional radio/ television in the household and distance to nearby health facilities had also effect on knowledge about danger signs. Mothers who had functional radio or television in the house hold and distance to nearby health facilities less than 1 h had more knowledgeable about danger signs as compared to their counterparts. This finding is in line with the study carried out in Aleta wondo district [18], Tsegedie district [27], Tanzania [28], Egypt [30] and Nepal [34]. This is the fact that hearing of mass media can be one of source of information. In addition, distance to health facilities was one of major obstacle to obtain maternal health services [34–36].

Maternal monthly income was other predictor that affects the level of knowledge about danger signs. Mothers with monthly income greater than 1000 Ethiopian birr had two times more knowledgeable about danger signs of postpartum as compared to those who have monthly income less than 300 Ethiopian birr. This finding is in line with the study carried out in Aleta wondo district [18], Tsegedie district [27], Tanzania [28], Egypt [30], Arba Minch district [31] and Nepal [34]. The reason for this association could be due to need of money to utilize health related services including transportation costs at any time they want.

## Limitation of the study

Since the study was cross sectional, it may not be strong to demonstrate direct cause and effect between dependent and independent variables.

## Conclusion

In this study, more than half of mothers were not knowledgeable about danger signs of childbirth and postpartum. This indicates that many mothers are more likely to delay in deciding to seek health care. Severe vaginal bleeding was the most common spontaneously mentioned danger signs during childbirth. The most important factors that affect knowledge of obstetric danger signs were educational status of mother, place of residence, place of delivery, exposure to hear of mass media and use of antenatal care. Based on the findings of the present study, it can be concluded that lack of awareness about danger signs of childbirth and postpartum were related to low level of education, home delivery, No ANC visit or only few number of focused ANC visit and less utilization of health service at health facilities. These factors were stressed and the need for plan to increase the awareness of public about danger signs. This information will help the services providers for improving the quality of obstetric care services through addressing those factors and strengthening of services through health information, communication and education.

## Supplementary information

**Additional file 1.** Questionnaires.

**Additional file 2.** Sampling Procedure.

## Data Availability

The data set used and/ or analyzed during the current study available from the corresponding author on reasonable request.

## Abbreviations

ANC: Ante natal care
AOR: Adjusted odds ratio
CI: Confidence interval
COR: Crude odds ratio
SNNPR: Southern nation nationalities peoples region
SPSS: Statistical package for social science
TV: Television
USD: United States dollar
