# Supplementary material for: Knowledge about child birth and postpartum obstetric danger signs and associated factors among mothers in Dale district, Southern Ethiopia
Source: BMC Pregnancy Childbirth. 2020 Jun 1;20:340. doi: 10.1186/s12884-020-02989-7 (PMC7268669; doi:10.1186/s12884-020-02989-7)
Supplement: Supplementary file 1 — Additional file 1. Questionnaires. [file 12884_2020_2989_MOESM1_ESM.pdf]

ANNEX I: QUESTIONNAIRES

ADAMA SCIENCE AND TECHNOLOGY UNIVERSITY

SCHOOL OF PUBLIC HEALTH

The Questionnaire was prepared to assess the level of knowledge about obstetric danger signs of childbirth and post partum among mothers who gave birth gave birth in the last 12 months prior to this survey in Dale district, Southern Ethiopia.

HOW TO APPROACH AND INTERVIEW THE HOUSEHOLD

You will be given the name of the kebele, the village and the house number that is going to be interviewed.

Once you get the household, you should get permission to enter in to the house. Do not enter in to the house without the permission of the household members.

- Introduce yourself by name, and explain the purpose of your visit.
- Tell the household member "Why" and "How" the household was selected for the study.
- Ask whether the eligible woman is present.
- If the eligible woman is there, briefly explain the purpose of the study by reading what was written on the questionnaire, and ask for her consent.
- If the eligible woman is not around, take an appointment for another visit.
- At the end of the interview, tell the respondent that it is the end of the interview, and thank the woman for giving her time and for her cooperation.

INTRODUCTION AND CONSENT:

Hello. My Name is ____________________ and I am part of a team of people who are carrying out a survey to assess the level of knowledge about obstetric danger signs of child birth and post partum among mothers who gave birth gave birth in the last 12 months prior to this survey in Dale district, (Show a letter of approval from Adama Science and technology university). We were very much appreciate you for your participation in this study. I would like to ask you some questions and it will take about 30 minutes. Your answers will remain confidential, and we will not be taking down your name or address, so your answers will be anonymous.

Participation in this survey is voluntary and you can choose not to answer any individual question or all of the questions. However, we hope that you will participate in this study since your views are important.

At this time, do you want to ask me anything about the study?

Do you agree to participate in this study? Yes …………. No…………

May I begin the interview now? 1. Yes 2. No

If no, go to the next house.

Thank you for your Cooperation.

IDENTIFICATION

01. Mother's ID number: ______________
02. Kebele ___________

PART I. SOCIO-DEMOGRAPHIC CONDITIONS

S.No	Questions and filters	Answers	Remark
101	Place of residence	1. Rural<br>2. Urban	
102	Age of the mother?	1. 15-19 years<br>2. 20-24 years<br>3. 25-29 years<br>4. 30-34 years<br>5. ≥35 years	
103	Current marital status of the mother?	1. Married<br>2. Divorced<br>3. Single<br>4. Widowed	
104	Religion of mother?	1. Protestant<br>2. Orthodox<br>3. Muslim<br>4. Catholic<br>5. Other(specify)__________	
105	Ethnic group of mother?	1. Sidama<br>2. Amhara<br>3. Oromo<br>4. Guraghe<br>5. Others(specify)__________	
106	Educational level of mother?	1. Unable to read and write<br>2. Read and Write<br>3. Primary education<br>4. Secondary education<br>5. College and above	

107	Occupation of the mother?	1. Housewife<br>2. merchant<br>3. Government employee’s<br>4. Private gainful worker<br>5. Other(specify)__________	
108	Monthly income in Ethiopian Birr	1. 100-300<br>2. 301-500<br>3. 500-1000<br>4. >1000	
109	Age of the husband?	1. 15-19 years<br>2. 20-24 years<br>3. 25-29 years<br>4. 30-34 years<br>5. ≥ 35 years	
110	Educational level of the husband	1. Illiterate<br>2. Read and Write<br>3. primary<br>4. Secondary education<br>5. College and above	
110	Occupation of the husband?	1. Farmer<br>2. Merchant<br>3. Government employee<br>4. No job<br>5. Other (specify)____________	
111	Monthly income in Ethiopian Birr	1. 100-300<br>2. 301-500<br>3. 500-1000<br>4. >1000	
112	Family Size	1. 1-3<br>2. 4-6<br>3. >6	
113	Do you have Radio/ Television in the household?	1. Yes<br>2. No	
114	Time taken to nearby health facilities	1. <1 hour<br>2. >= 1 hour	

PART II: MATERNAL AND OBSTETRIC CONDITION

S.No	Questions and filters	Answers	Remark
201	Age at first pregnancy	1. 15-19 years<br>2. 20-29 years<br>3. 30 and above	
202	How many times in total you became pregnant?	1. 1<br>2. 2 – 3<br>3. 4 and above	
203	How many times in total you gave birth?	1. 1<br>2. 2-3<br>3. 4 and above	
204	How many of your pregnancies resulted in a baby that was born Alive?	1. 0<br>2. 1<br>3. 2<br>4. 3 and above	
205	How many of your pregnancies resulted in a baby that was born Dead?	1. 0<br>2. 1<br>3. 2<br>4. 3 and above	
206	Are you ever-used antenatal care during last pregnancy?	1. Yes<br>2. No →	Skip to 208
207	How many times you visit antenatal care during last pregnancy?.	1. 1 visit<br>2. 2-3 visit<br>3. 4 visit<br>4. 4 and above Visit	
208	Where you deliver previous birth?	1. Home<br>2. Health institution<br>3. Other specify_____________	
209	Who were final decision maker on place of delivery during last pregnancy?	1. Self<br>2. Husband<br>3. Relatives<br>4. Other----------------------	

PART III: KNOWLEDGE

S.No	Questions and filters	Answers	Remark
301	Do you know any/ some serious health problem/s that can occur during pregnancy that could endanger the life of a pregnant woman?	1. Yes<br>2. No →	 Skip to 303
302	Can you mention them?	1. Vaginal bleeding<br>2. Severe headache<br>3. Blurred vision<br>4. Convulsions<br>5. Swollen hands/face.<br>6. High fever<br>7. Loss of consciousness<br>8. pesistant nausea &Vomiting<br>9. Severe weakness<br>10. Severe abdominal pain<br>11. Accelerated/reduced fetal movement<br>12. Water breaks without labor<br>13. Other (Specify)-------------	
303	Do you know any/some serious health problem/s that can occur during labor and<br>Child birth? That could endanger the life of a pregnant woman?	1. Yes<br>2. No →	 Skip to 305
304	Can you mention them?	1. Severe vaginal bleeding<br>2. Severe headache<br>3. Convulsions<br>4. High fever<br>5. Loss of consciousness<br>6. Labor lasting>12hours<br>7. Placenta not delivered 30 minutes after delivery<br>8. Other (Specify)_____________	

305	Do you know any/some serious health problem/s that can occur during the first 42<br>Days after birth that could endanger the life of the women?	1. Yes<br>2. No →	Skip to 307
306	Can you mention them?	1. Severe vaginal bleeding<br>2. Severe headache<br>3. Blurred vision.<br>4. Convulsions<br>5. Swollen hands/face<br>6. High fever<br>7. Loss of consciousness.<br>8. Difficulty breathing.<br>9. Severe weakness<br>10. Malodorous vaginal discharge<br>11. Others (specify)____________	
307	From where you get information of danger signs	1. Antinatal care<br>2. Pevious delivery experiance<br>3. General awareness in the community<br>4. Health education given at Health facilities<br>5. Heard of mass media(radio/TV)<br>6. Other (specify)-----------------------	
