# Supplementary material for: Knowledge about child birth and postpartum obstetric danger signs and associated factors among mothers in Dale district, Southern Ethiopia
Source: BMC Pregnancy Childbirth. 2020 Jun 1;20:340. doi: 10.1186/s12884-020-02989-7 (PMC7268669; doi:10.1186/s12884-020-02989-7)
Supplement: Supplementary file 2 — Additional file 2. Sampling Procedure. [file 12884_2020_2989_MOESM2_ESM.pdf]

Sampling Procedure

Multistage sampling was used to select the study subjects. First, all the kebeles /sub-districts in the district was stratified into urban and rural. Then 2 out of 6 urban and 11 out of 36 rural Kebeles were randomly selected. The calculated sample size was proportionally allocated to urban (n=120) and rural (n=662) according to their number of households. Then, sampling frames of households was prepared for each kebele in collaboration with the administrators of respective kebeles. Households with a woman who gave birth in the last 12 months prior to the survey were selected and grouped into the village and the villages were selected by using simple random sampling. For selecting the study participants, eligible women who found in the selected villages were the part of the study until sample size allocated for each kebele was enough. Whenever more than one eligible respondents were found in the same selected household, only one respondent was chosen by lottery method.

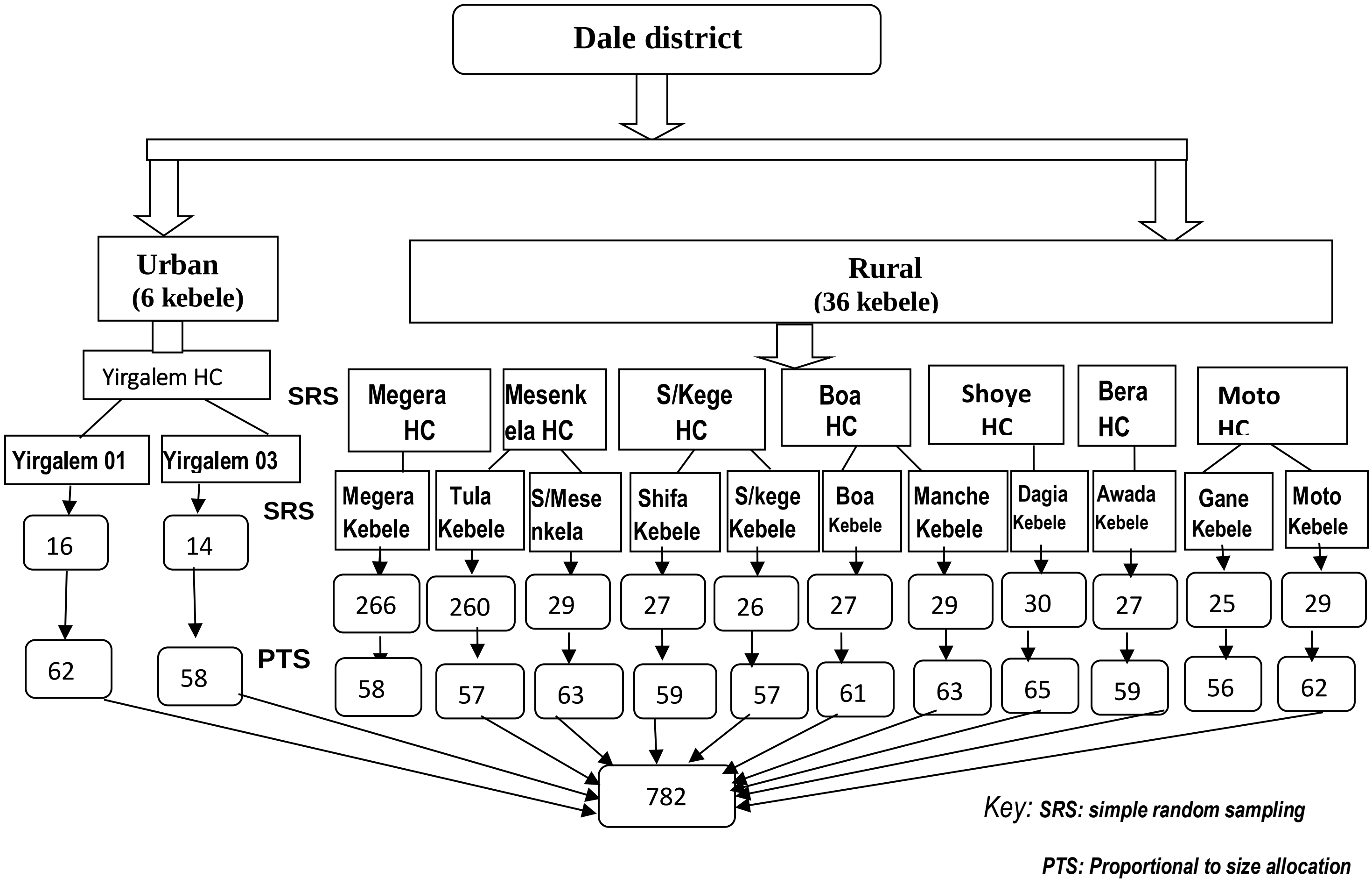

Fig.2. shows the data collection Procedure
